# Supplementary material for: Emergent Rate Laws for Collective Lying–Standing Transitions
Source: ACS Phys Chem Au. 2026 Jun 9;6(4):815–26. doi: 10.1021/acsphyschemau.6c00029 (PMC13397434; doi:10.1021/acsphyschemau.6c00029)
Supplement: Supplementary file 1 [file pg6c00029_si_001.pdf]

Supporting Information

Emergent Rate Laws for Collective Lying–Standing Transitions

Anna Werkovits[1,†], Simon B. Hollweger[1,†], Oliver T. Hofmann[1,*]

[1] *Institute of Solid State Physics, Graz University of Technology, 8010 Graz, Austria*

† *A.W. and S.B.H. contributed equally to this work.*

*Email: o.hofmann@tugraz.at

Contents

S1 Modeling details . . . 1
S2 kMC Sampling Details . . . 4
S3 Irreversible Power-Law Two-State Approximation . . . 4
S4 Apparent Reaction Orders . . . 6
S5 Arrhenius Plots . . . 7
S6 Derivation of effective diffusion-induced stabilization . . . 8

Supporting Information

S1 Modeling details

Figure S1 visualizes the kMC representation of model systems with different footprint ratios. The surface is modeled as a square lattice with lattice constant l_{uc}, which defines the smallest unit of surface area accessible to the adsorbate. The lying–standing footprint ratio f determines how many kMC lattice units are spanned by the molecular footprint. In the discrete kMC representation, this is implemented by mapping the footprint areas onto lattice units: Lying molecules occupy $f \times f$ contiguous lattice cells, while standing molecules occupy either $f \times 1$ or $1 \times f$ lattice cells, reflecting the two possible orientations of standing molecules on the surface. The footprint ratio thus directly determines how many lattice units are occupied by an adsorbate in each orientation and how vacancies generated during reorientation can be stabilized by adsorption. Further details on the kMC representation can be found in the Supporting Information of previous work.[1]

Figure S2 illustrates the geometric definition of the on-surface processes for the reference model system (footprint ratio $f = 2$, molecular size $l_{\mathrm{L}} = 6.8\,\text{Å}$, and lying adsorption energy $E_{\mathrm{ads,L}} = -2.4\,\mathrm{eV}$). Lying molecules are shown as blue squares, while standing molecules are shown as orange rectangles in horizontal or vertical orientation. Initial positions are indicated by filled shapes and final positions by hatched shapes, with arrows marking the possible transition directions. Model systems with larger footprint ratios behave

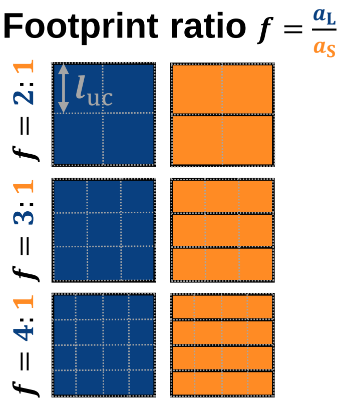

Figure S1: kMC representation of model systems with footprint ratios $f = 2$, 3, and 4. This figure extends Figure 6 of the main text, where footprint areas of molecules in lying (a_L) and standing (a_S) orientations are denoted. For a footprint ratio $f = 2$, the lying molecule is represented by a 2×2 square of the kMC lattice (square lattice with lattice constant l_{uc}), and by 3×3 and 4×4 squares for $f = 3$ and 4, respectively. Standing molecules occupy either $1 \times f$ or $f \times 1$ areas of the kMC lattice, corresponding to the two adsorption orientations of standing molecules (horizontal and vertical).

analogously: Diffusion proceeds in steps of one kMC lattice unit, and reorientation always occurs via the edges.

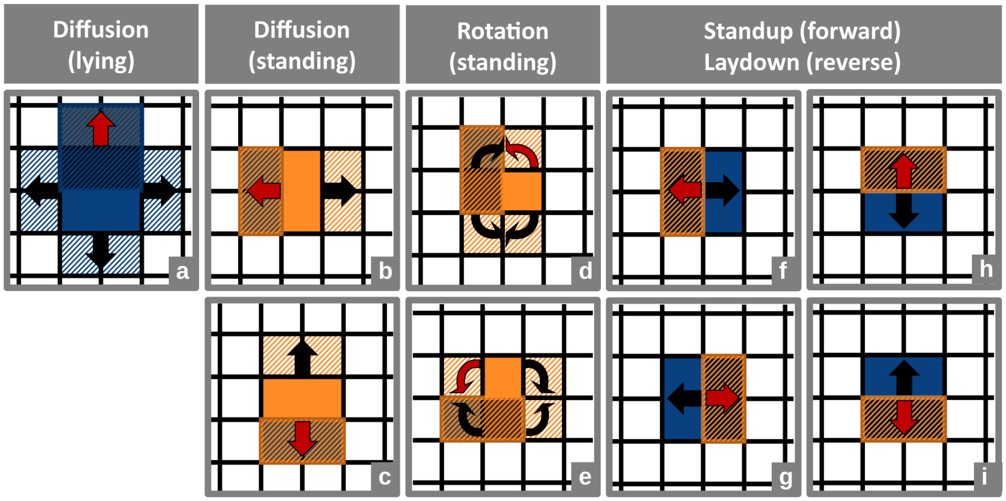

Figure S2: Definition of on-surface processes for the reference model system (footprint ratio $f = 2$) showing the three possible adsorption geometries lying, standing horizontal, and standing vertical. Shown are diffusion in lying (a) and standing (b-c) orientations, rotation in standing orientation (d-e), and reorientation from lying to standing (forward transition; f-i), referred to as stand-up, with the reverse transition termed lay-down. Initial adsorption sites are visualized as filled blue squares or orange rectangles, respectively, while final adsorption sites are shown with hatched shapes. Arrows indicate the transition directions. Only forward transitions are shown explicitly, whereas reverse transitions correspond to the inverted pathways. This schematic representation is adapted from the Supporting Information of ref [1].

The lattice constant of the reference system ($f_{ref} = 2$) is $l_{uc,ref} = 3.4$ Å, adopted from the coarse-grained kMC model of TCNE/Cu(111) introduced previously.[1] Note that this is an approximation arising from the use of a square lattice instead of the native hexagonal Cu(111) lattice, whose lattice constant is 3.6 Å. All simulations are designed such that, irrespective of the footprint ratio, $n_L \times n_L = 12 \times 12 = 144$ molecules

in the lying orientation fully cover the surface. The supercell size n_{sc} (number of repeating unit cells per dimension) is therefore given by

$$n_{\mathrm{sc}} = n_{\mathrm{L}} \cdot f. \tag{14}$$

For footprint ratios $f = 2$, 3, and 4, this results in supercell sizes of 24×24, 36×36, and 48×48, respectively. The lattice constants l_{uc} for larger footprint ratios $f = 3$ and 4 follow naturally Equation 15. Therein, the lattice constant is obtained by dividing the size of the lying molecule l_{L} by the footprint ratio f. This procedure yields lattice constants of 2.27 Å and 1.70 Å for footprint ratios of $f = 3$ and 4, respectively.

$$l_{\mathrm{uc}} = \frac{l_{\mathrm{L}}}{f} \tag{15}$$

In the dataset where the molecular size l_{L} is varied, the lattice constant l_{uc} is adjusted according to Equation 15. The molecular size itself is varied in (half-)integer multiples of the lattice constant of the reference system, $l_{\mathrm{uc,ref}} = 3.4$ Å. The resulting lattice constants are summarized in Table S1.

Table S1: Lattice constants l_{uc} for varying molecular sizes l_{L} and footprint ratios f.

l_{L} / Å	l_{uc} ($f = 2$) / Å	l_{uc} ($f = 3$) / Å	l_{uc} ($f = 4$) / Å
$2.0 \times l_{\mathrm{uc,ref}} = 6.8$	3.40	2.27	1.70
$2.5 \times l_{\mathrm{uc,ref}} = 8.5$	4.25	2.83	2.13
$3.0 \times l_{\mathrm{uc,ref}} = 10.2$	5.10	3.40	2.55
$3.5 \times l_{\mathrm{uc,ref}} = 11.9$	5.95	3.97	2.98
$4.0 \times l_{\mathrm{uc,ref}} = 13.6$	6.80	4.53	3.40
$4.5 \times l_{\mathrm{uc,ref}} = 15.3$	7.65	5.10	3.82

To address the time-scale disparity arising from rate constants differing by many orders of magnitude, a time-acceleration algorithm is employed in the kMC simulations.[2, 3] Because the number of standing molecules differs for models with different footprint ratios, the buffer parameter is adjusted to approximately the number of lattice sites in the corresponding simulation cell, rounded up to the next thousand. For the threshold, execution, and sampling parameters, default values were found to be sufficient. The explicit parameters are summarized in Table S2.

Table S2: Time-acceleration parameters for kMC simulations with varying footprint ratios f.

	$f = 2$	$f = 3$	$f = 4$
`buffer_parameter`	1000	2000	3000
`threshold_parameter`	0.2	0.2	0.2
`execution_parameter`	200	200	200
`sampling_steps`	20	20	20

S2 kMC Sampling Details

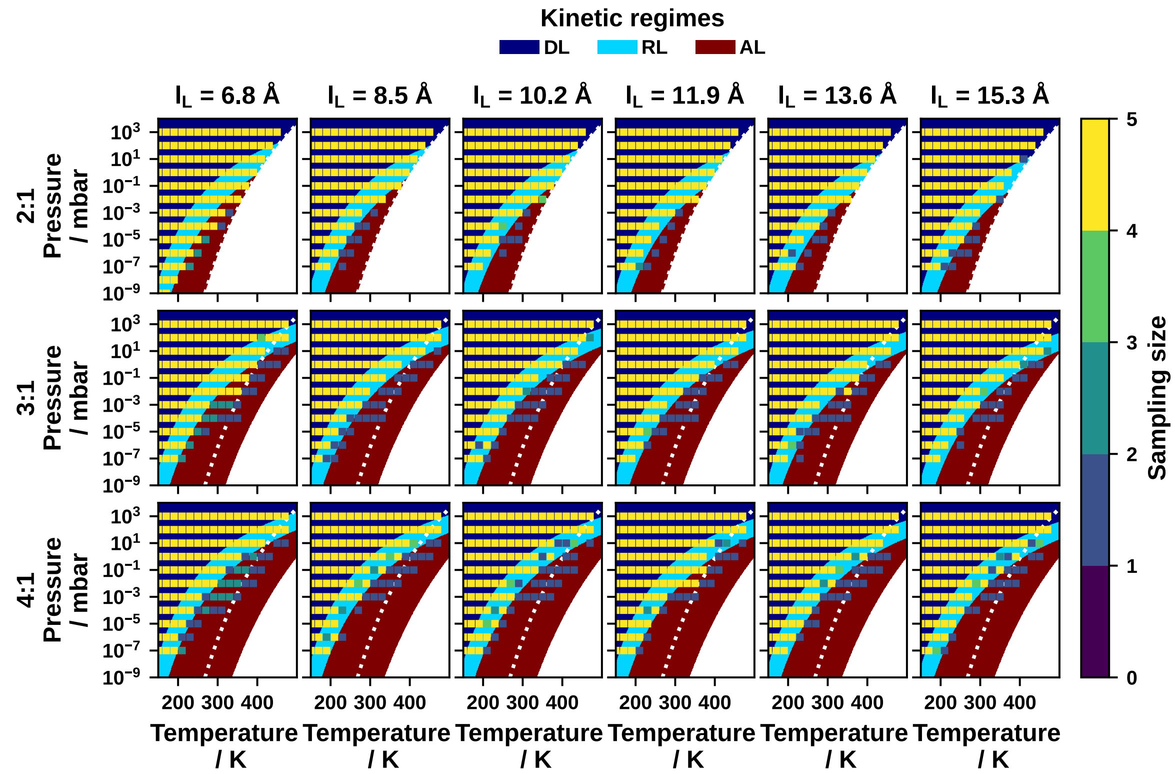

Figure S3: Kinetic Monte Carlo sampling size of temperature–pressure points. Color coding indicates the number of independent simulations performed at identical temperature T, pressure p, adsorbate size l_{L} (columns), and footprint ratio f (2:1, 3:1 and 4:1; rows). A target sampling size of five simulations was aimed for but is not reached in all cases due to computational cost. The background delineates the kinetic regimes (diffusion-limited, DL; reorientation-limited, light blue; adsorption-limited, dark red) and the thermodynamic stability region of the lying phase (white).

S3 Irreversible Power-Law Two-State Approximation

Validity near the thermodynamic lying–standing phase boundary

As the irreversibility assumption is expected to be least accurate in the vicinity of the thermodynamic lying-standing phase boundary, we explicitly assess this region. In this regime, lying and standing domains coexist at long times due to phase equilibrium, and a finite backward flux is expected. Even under these conditions, the collective reorientation rate constants remain well captured by the IPL2SA framework. Deviations occur primarily in the extracted reaction order α, reflecting the increasing influence of reversible fluctuations near coexistence. Within the irreversible two-state ansatz, this residual reversibility is effectively absorbed into the fitted reaction order, while the model drives the standing coverage toward $\theta_{\mathrm{S}} \to 1$, although strictly $\theta_{\mathrm{S}} < 1$ at coexistence due to phase balancing between lying and standing domains. In our analysis we are aware of these deviations, that are rather recognized as methodological artifacts than as indications of a breakdown of the effective description.

Quality of IPL2SA Fits

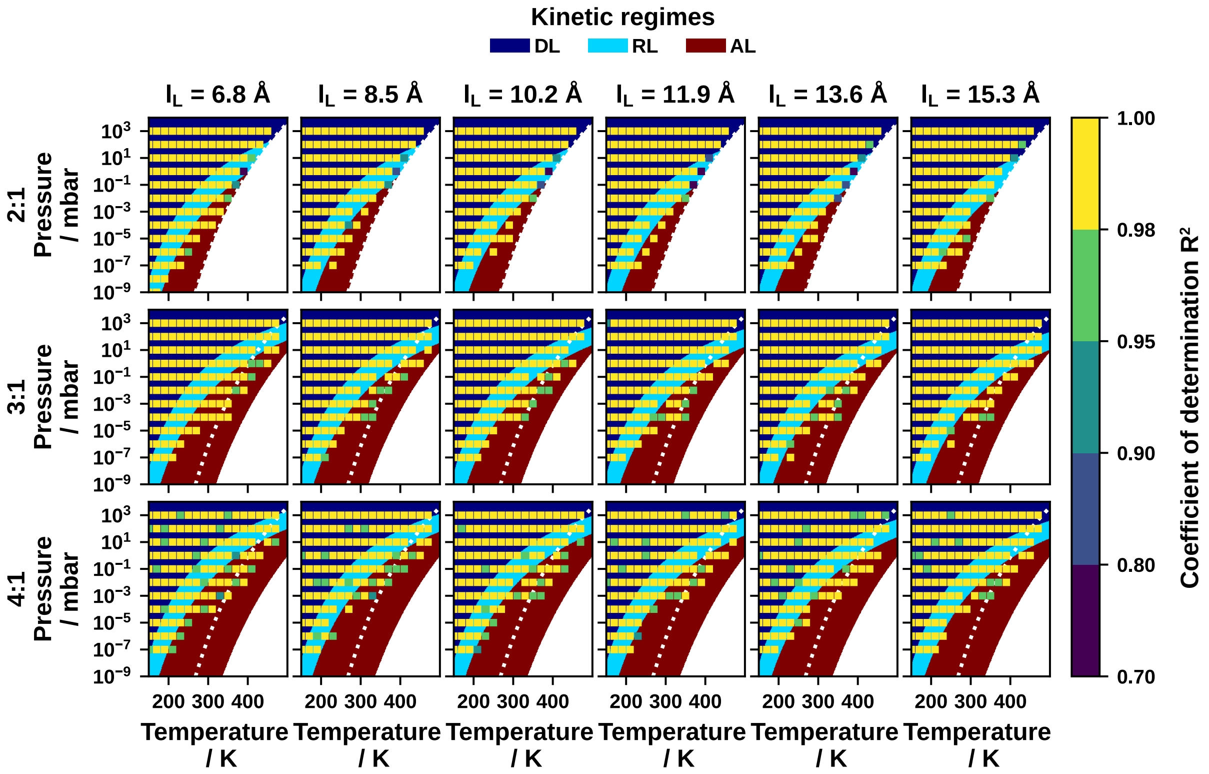

Figure S4: Quality of the *Irreversible Power-Law Two-State Approximation (IPL2SA)* for the temperature–pressure points sampled by kinetic Monte Carlo. The color scale quantifies the quality using the coefficient of determination R^2 for every combination of footprint ratios f (2:1, 3:1 and 4:1) and adsorbate size l_{L}. It is computed by comparing coverages from kinetic Monte Carlo (kMC) snapshots (approximately logarithmically spaced in time) with the corresponding IPL2SA predictions. The fit and R^2 evaluation are restricted to the most relevant regime of the lying–standing transition, namely the time interval where the standing coverage fraction θ_{S} lies within 0.5 to 0.95. The background delineates the kinetic regimes (diffusion-limited, dark blue; reorientation-limited, light blue; adsorption-limited, dark red) and the thermodynamic stability region of the lying phase (white).

S4 Apparent Reaction Orders

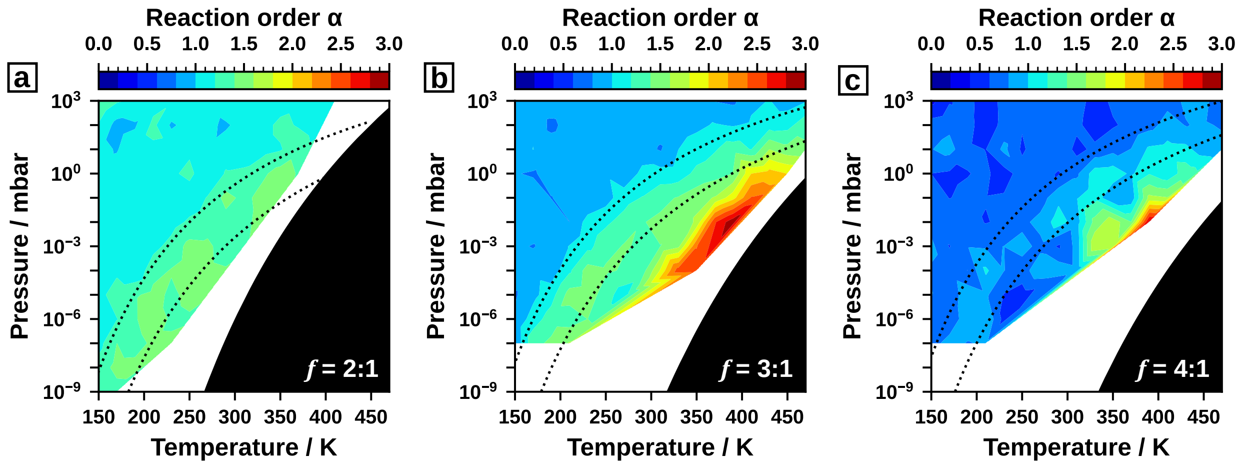

Figure S5: Pressure–temperature diagrams of the apparent reaction order α for model systems with increasing footprint ratios: (a) **2:1**, (b) **3:1**, and (c) **4:1**. α quantifies how strongly the transition depends on coverage and accordingly indicates steric effects like enhanced cooperativity ($\alpha < 1$) and inhibition ($\alpha > 1$). Note, that this is not a quantity from one microscopic process - rather it is an effective quantity from a collective transition obtained by the *IPL2SA* via fitting (Equation 12 and 13). An identical color scale is used for all panels to enable direct comparison. Boundaries between diffusion-limited (DL), reorientation-limited (RL), and adsorption-limited (AL) regimes are indicated by dotted lines, and the corresponding α ranges are annotated. The thermodynamic phase diagram is shown in the background for reference, highlighting regions where standing (white) or lying (black) molecules are thermodynamically stable.

S5 Arrhenius Plots

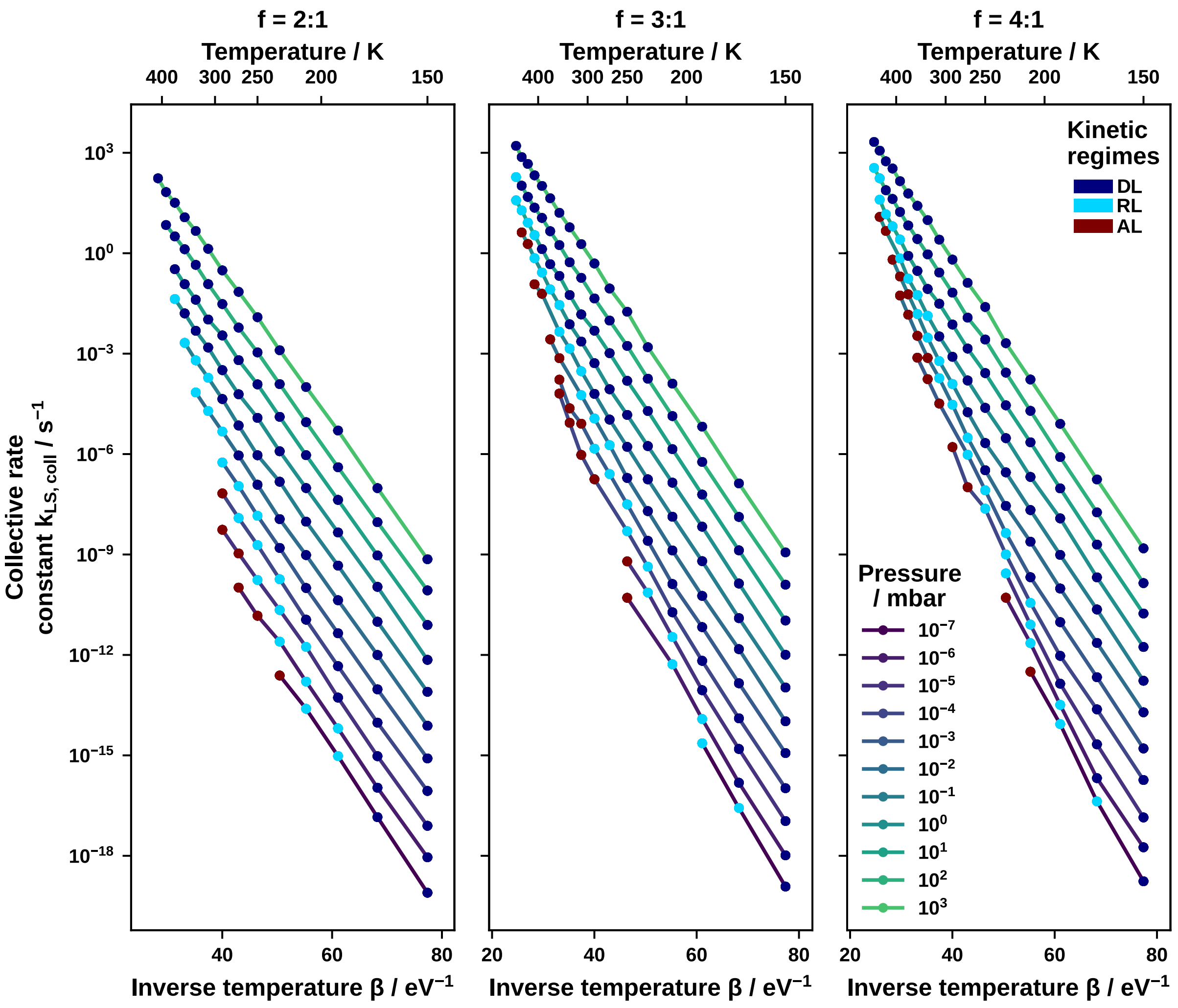

Figure S6: Arrhenius plots for footprint ratios $f = 2{:}1$, 3:1, and 4:1 at fixed adsorbate size $_{\mathrm{L}} = 6.8$ Å. For each footprint ratio, collective rate constants are shown as a function of inverse temperature $\beta = (k_{\mathrm{B}}T)^{-1}$ (bottom axis) at different pressures (lines; see pressure legend). The corresponding temperature T is indicated on the upper axis. Temperature–pressure points are assigned to their respective kinetic regimes, as indicated by the color coding (see legend).

S6 Derivation of effective diffusion-induced stabilization

In the main text, diffusion of lying molecules is shown to stabilize newly formed standing molecules by removing the standing molecule from the vacancy created during reorientation (cf. States $*_i$ in Figure 5). In this section, the corresponding correction to the local two-step reorientation model is derived in terms of an effective probability that a standing molecule can fall back into the lying configuration.

Probability for back-reorientation

Back-reorientation (S→L) requires that (i) a vacancy exists and (ii) the vacancy is adjacent to the standing molecule such that the lying footprint can be accommodated. We therefore write the probability that the back-reorientation condition is met as

$$p_{\mathrm{SL}} = p_{\mathrm{vac}} \cdot p_{\mathrm{vac+S}}, \tag{16}$$

where p_{vac} denotes the probability that a vacancy exists, and $p_{\mathrm{vac+S}}$ the conditional probability that a vacancy is located next to a standing molecule (irrespective of the remaining local environment).

Complementary formulation via vacancy blocking by lying molecules

The probability $p_{\mathrm{vac+S}}$ can be expressed via its complementary event, namely that the site adjacent to the standing molecule is occupied by a lying molecule, which sterically blocks immediate back-reorientation. Defining $p_{\mathrm{vac+L}}$ as the probability that a lying molecule occupies the relevant neighboring site, one obtains

$$p_{\mathrm{vac+S}} = 1 - p_{\mathrm{vac+L}}. \tag{17}$$

Within the effective description, $p_{\mathrm{vac+L}}$ is approximated by the ratio of the rate for establishing a blocking configuration (occupation by a lying molecule) and the total rate of all competing processes that prevent immediate back-reorientation. Concretely, the blocking event is governed by diffusion of lying molecules with rate constant k_{LL}. Competing processes comprise (i) adsorption of standing molecules into any of the n_{vac} adsorption-enabled vacancies with rate $n_{\mathrm{vac}} k_{\mathrm{ads,S}}$, and (ii) further lying-diffusion events that generate additional neighboring configurations, captured by an effective multiplicity factor ω multiplying k_{LL}. This yields

$$p_{\mathrm{vac+L}} = \frac{k_{\mathrm{LL}}}{k_{\mathrm{LL}} + n_{\mathrm{vac}} k_{\mathrm{ads,S}} + \omega k_{\mathrm{LL}}}, \tag{18}$$

and therefore

$$p_{\mathrm{vac+S}} = 1 - \frac{k_{\mathrm{LL}}}{k_{\mathrm{LL}} + n_{\mathrm{vac}} k_{\mathrm{ads,S}} + \omega k_{\mathrm{LL}}}. \tag{19}$$

Effective back-reorientation propensity and geometric prefactor

Using Eq. 16, the effective propensity for back-reorientation scales with p_{SL}, such that the corresponding loss term can be written as

$$\frac{\mathrm{d}p_1}{\mathrm{d}t} \propto n_{\mathrm{SL}}\, k_{\mathrm{SL}}\, p_{\mathrm{SL}} = n_{\mathrm{SL}}\, k_{\mathrm{SL}}\, p_{\mathrm{vac}}\, p_{\mathrm{vac+S}}, \tag{20}$$

Consequently, the diffusion-induced stabilization can be absorbed into the geometric prefactor γ of the effective collective rate expression by renormalizing the probability for back-reorientation. In the notation of the main text this yields

$$\gamma = \frac{n_{\mathrm{vac}}\, n_{\mathrm{LS}}}{n_{\mathrm{SL}}\, p_{\mathrm{vac+S}}} \quad \text{with} \quad p_{\mathrm{vac+S}} = 1 - \frac{k_{\mathrm{LL}}}{k_{\mathrm{LL}} + n_{\mathrm{vac}} k_{\mathrm{ads,S}} + \omega k_{\mathrm{LL}}}. \tag{21}$$

Equations 19–21 provide the diffusion-corrected contribution of vacancy–molecule decoupling to the effective geometric factor used to rationalize the regime-dependent collective kinetics.

References

(1) Werkovits, A.; Hollweger, S. B.; Niederreiter, M.; Risse, T.; Cartus, J. J.; Sterrer, M.; Matera, S.; Hofmann, O. T. Kinetic Trapping of Charge-Transfer Molecules at Metal Interfaces. *The Journal of Physical Chemistry C* **2024**, *128*, 3082–3089, DOI: 10.1021/acs.jpcc.3c08262.

(2) Dybeck, E. C.; Plaisance, C. P.; Neurock, M. Generalized Temporal Acceleration Scheme for Kinetic Monte Carlo Simulations of Surface Catalytic Processes by Scaling the Rates of Fast Reactions. *Journal of Chemical Theory and Computation* **2017**, *13*, 1525–1538, DOI: 10.1021/acs.jctc.6b00859.

(3) Andersen, M.; Plaisance, C. P.; Reuter, K. Assessment of mean-field microkinetic models for CO methanation on stepped metal surfaces using accelerated kinetic Monte Carlo. *The Journal of Chemical Physics* **2017**, *147*, 152705, DOI: 10.1063/1.4989511.
